# Variability of Paranasal Sinus Pneumatization in the Absence of Sinus Disease

**DOI:** 10.31486/toj.19.0053

**Published:** 2020

**Authors:** Michael J. Marino, Charles A. Riley, Eric L. Wu, Jacqueline E. Weinstein, Noah Emerson, Edward D. McCoul

**Affiliations:** ^1^ Department of Otorhinolaryngology, Mayo Clinic, Phoenix, AZ; ^2^ Division of Otolaryngology, Fort Belvoir Community Hospital, Fort Belvoir, VA; ^3^ Department of Otolaryngology–Head and Neck Surgery, Tulane University School of Medicine, New Orleans, LA; ^4^ Division of Pediatric Otolaryngology–Head and Neck Surgery, Benioff Children's Hospital, University of California, San Francisco, San Francisco, CA; ^5^ Department of Radiology, Ochsner Clinic Foundation, New Orleans, LA; ^6^ Department of Otorhinolaryngology, Ochsner Clinic Foundation, New Orleans, LA; ^7^ The University of Queensland Faculty of Medicine, Ochsner Clinical School, New Orleans, LA

**Keywords:** *Anatomic variation*, *paranasal sinuses*, *tomography–x-ray computed*

## Abstract

**Background:** Paranasal sinus pneumatization is a complex process, and numerous computed tomography (CT) studies document developmental variations in the setting of underlying sinus disease. The purpose of this study was to investigate variation in paranasal sinus pneumatization in a population of nondiseased subjects using a metric validated for tracking individual anatomic variants as well as total sinus volume.

**Methods:** A total of 591 consecutive sinus and maxillofacial CT scans were considered for study inclusion. After patients with inflammatory sinus or respiratory disease were excluded, 323 CT scans were scored using the Assessment of Pneumatization of the Paranasal Sinuses (APPS) instrument, and relevant demographic data were recorded for each scan. APPS findings were compared according to demographic characteristics and laterality.

**Results:** Laterality and sex were associated with differences in paranasal sinus pneumatization in a nondiseased population. Based on APPS score, the left side (4.95) was more pneumatized than the right (4.74, *P*=0.006), and males (10.16) were more extensively pneumatized than females (9.18, *P*=0.005). We found no correlation of age with sinus pneumatization (ρ=0.025). The probability of perceptible asymmetry in any given individual's paranasal sinus pneumatization was 69%, and the probability of left-sided dominance was 53%.

**Conclusion:** Substantial anatomic variation exists in paranasal sinus anatomy, even among patients without sinus disease. Significant differences are found between males and females and between the left and right sides. Continued systematic research of paranasal sinus anatomy may facilitate a standard for CT sinus assessment that will aid clinician evaluation of anatomic variation and surgical decision making.

## INTRODUCTION

Paranasal sinus pneumatization is a complex and incompletely understood process. Multiple reports associate the anatomic variations seen on sinus computed tomography (CT) with specific sinonasal diseases. Paranasal sinus hypoplasia has been well established in cystic fibrosis,^1-4^ and similar variations have been reported in patients with primary ciliary dyskinesia and Kartagener syndrome.^5^ Variations of paranasal sinus pneumatization in the setting of chronic rhinosinusitis have been less clear. Studies have reported decreased maxillary sinus pneumatization,^6,7^ increased frontal sinus pneumatization,^8^ or no differences compared to patients without chronic rhinosinusitis.^9^ Multiple theories have been proffered to account for the differences in paranasal sinus pneumatization, including the effect of chronic sinus inflammation and infection,^9,10^ genetic mechanisms,^1,3,4^ regional blood flow anamolies,^11,12^ and increased serum erythropoietin.^11^

In addition to the potential associations between differential sinus pneumatization and clinical disease states, nondiseased patients also appear to have considerable anatomic variation. The presence of at least a single sinonasal anatomic variant has been reported to be 64.0% to 99.8% in studies of patients with and without mucosal sinus disease.^13-15^ As many as 52 bony or air cell variants have been identified, and up to 41% were without apparent impact on clinical disease, endoscopic vision, or exposure of critical structures.^15^ Nevertheless, differing definitions of specific anatomic variations remain a concern for reliable identification.^16^ Defining the degree of paranasal sinus anatomic variation and overall pneumatization using a validated method may be useful for clinicians in understanding baseline variability and could also help to identify intrinsic patient characteristics that are associated with differing pneumatization or specific variations.

**Figure 1.**
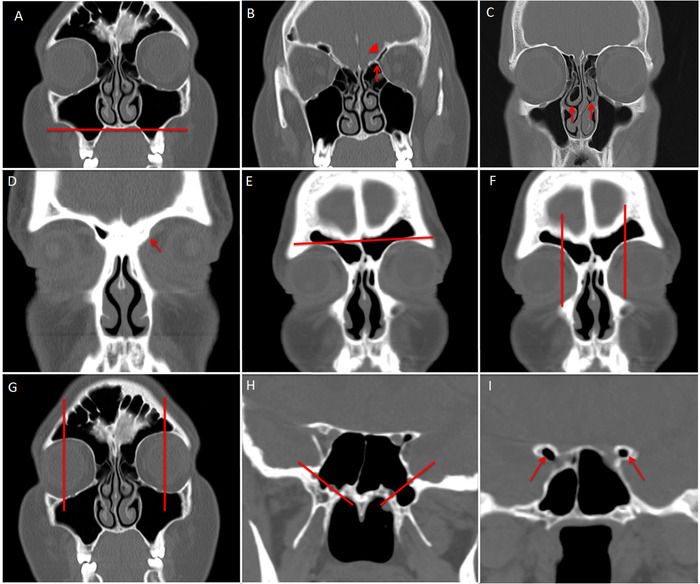
**(A) The floor of the maxillary sinus extends inferior to the floor of the nasal cavity, corresponding with APPS item 1. (B) Supraorbital air cell (arrowhead) is present superior to the anterior ethmoid artery (arrow), corresponding with APPS item 2. (C) Middle turbinate concha bullosa is present (arrows), corresponding with APPS item 3. (D) The frontal sinus is absent (arrow), corresponding with APPS item 4. (E) The frontal sinus extends superior to the supraorbital rim, corresponding with APPS item 5. (F) The lateral wall of the frontal sinus extends lateral to the medial edge of the globe, corresponding with APPS item 6. (G) The lateral wall of the frontal sinus extends lateral to the midpupillary line, corresponding with APPS item 7. (H) The lateral wall of the sphenoid sinus extends beyond to the V_2_-VN line, corresponding with APPS item 8. (I) The anterior clinoid process is pneumatized (arrows), corresponding with APPS item 9.** APPS, Assessment of Pneumatization of the Paranasal Sinuses; V_2_, maxillary nerve canal, VN, Vidian nerve canal.

The Assessment of Pneumatization of the Paranasal Sinuses (APPS) score was introduced as a radiographic instrument for tracking anatomic variation on sinus CT and is validated for interrater and intrarater reliability.^17^ The APPS instrument evaluates for the presence of 9 variants bilaterally (Table 1, Figure 1), and each item present is assigned a score of 1 for a total possible score range of 0 to 18. The total APPS score has been shown to correlate strongly with the total sinus volume as calculated by 3-dimensional volumetric analysis of sinus CT.^18^

**Table 1.** Assessment of Pneumatization of the Paranasal Sinuses Items

Item	Anatomic Variant
1	Maxillary floor inferior to nasal floor
2	Supraorbital cell (air cell superior to anterior ethmoid artery)
3	Middle turbinate concha bullosa present
4	Frontal sinus present
5	Superior frontal sinus wall superior to supraorbital rim
6	Lateral frontal sinus wall lateral to medial edge of globe
7	Lateral frontal sinus wall lateral to midpupillary line
8	Lateral sphenoid sinus wall lateral to V_2_-VN line
9	Anterior clinoid process pneumatized

V_2_, maxillary nerve canal; VN, Vidian nerve canal.

For this study, we used the APPS score as a validated metric to investigate the presence of individual anatomic sinus variants in a nondiseased population. Also, because the APPS score correlates with total sinus volume, we compared overall paranasal sinus pneumatization by demographic characteristics and laterality. Understanding anatomic and pneumatization variants may facilitate clinician evaluation of baseline paranasal sinus variation and identification of influencing factors.

## METHODS

Approval for this study was obtained from the Ochsner Clinic Foundation Institutional Review Board. A total of 591 sinus and maxillofacial CT scans performed at the senior author's primary institution between January 1, 2010, and August 15, 2015, were evaluated for APPS and Lund-Mackay scores. The Lund-Mackay score is a validated instrument for measuring the degree of sinus opacification.^17,19^

Total APPS scores (ranging from 0 to 18) were used to assess overall paranasal sinus pneumatization, and laterality-based comparisons were performed using unilateral APPS scores (ranging from 0 to 9). Patients with inflammatory sinus or respiratory disease, including chronic rhinosinusitis, allergic rhinitis, recurrent acute rhinosinusitis, cystic fibrosis, asthma, obstructive sleep apnea/sleep-disordered breathing, and maxillofacial fractures were excluded from the study. These diagnoses were ascertained retrospectively from the medical record. Also, only patients 13 years or older were included in this study, the age at which sinus pneumatization is presumed to be complete.^20^ A nondiseased population of 323 CT scans was included for analysis. The scans had been typically obtained for evaluation of suspected facial trauma, headache, or other nonsinonasal complaints. Radiographic scores and relevant demographic data, including age at the date of CT acquisition, ethnicity, and sex, were stored in a secure, web-based Research Electronic Data Capture v.6.6.2 (REDCap Vanderbilt University Medical Center) database for management and analysis.

The nondiseased population was analyzed for differences in paranasal sinus pneumatization according to demographic characteristics and laterality. The frequency of individual anatomic variants was also analyzed. Paired continuous variables were analyzed using paired *t* tests, while unpaired data were compared using independent two-sample *t* tests. Analyses comparing more than 2 groups simultaneously were performed using one-way analysis of variance. Chi-square test was used to compare categorical variables. Correlation analysis was performed using the Spearman rho. Common language effect size index was used to determine effect size as a probability.^21^ *P* values <0.05 were considered significant. Statistical analysis was performed using SAS software v.9.3 (SAS Institute Inc.).

## RESULTS

**Figure 2.**
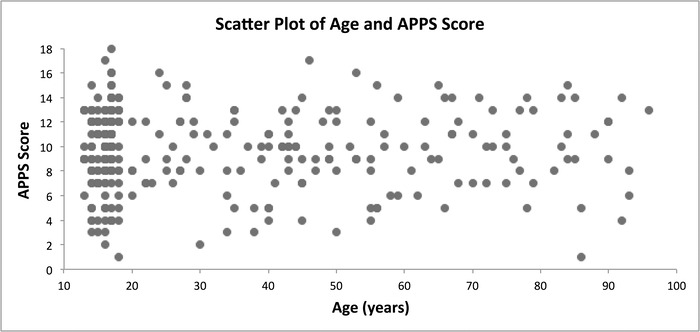
**Scatter plot of age and Assessment of Pneumatization of the Paranasal Sinuses (APPS) score for all computed tomography scans included in the study (n=323) shows no correlation between these parameters (ρ=0.025, *P*=0.657).**

The demographic characteristics and mean radiographic scores of the entire study population are presented in Table 2. In the comparison of the extent of paranasal sinus pneumatization by sex, males had statistically significant increased pneumatization compared to females (*P*=0.005, Table 3). The anatomic variation among ethnic groups did not reach statistical significance (*P*=0.148), and the extent of sinus pneumatization was not correlated with age (ρ=0.025, *P*=0.657, Figure 2).

**Table 2.** Demographic and Radiographic Characteristics of the Study Population

Variable	All Patients (n=323)
Age, years, mean ± SD	32.6 ± 22.9
Sex, n (%)	
Male	166 (51.4)
Female	157 (48.6)
Ethnicity,^a^ n (%)	
Caucasian/white (non-Hispanic)	201 (62.2)
African American/black	95 (29.4)
Latinx/Hispanic	18 (5.6)
APPS score, mean ± SD	9.68 ± 3.16
Lund-Mackay score, mean ± SD	2.21 ± 2.50

^a^Nine patients reported a different ethnic group or declined to respond.

APPS, Assessment of Pneumatization of the Paranasal Sinuses.

**Table 3.** Paranasal Sinus Pneumatization According to Sex

	Age, years, mean ± SD	APPS Score, mean ± SD
Male (n=166)	27.7 ± 19.3	10.16 ± 3.02
Female (n=157)	37.7 ± 25.3	9.18 ± 3.24
*P* Value	<0.001	0.005

APPS, Assessment of Pneumatization of the Paranasal Sinuses.

In the comparison of paranasal sinus pneumatization according to laterality, the left side had statistically significant increased pneumatization compared to the right side (*P*=0.006) (Table 4). This difference between sides increased when the more extensively pneumatized side (either left or right) was compared to the less pneumatized side (*P*<0.001). The left side more frequently had increased pneumatization (36.5% of subjects) vs the right side (30.7% of subjects). Both sides were symmetric in 32.8% of cases. Asymmetry, in general, was more frequent than symmetry between the sides (*P*<0.001). According to the common language effect size index, the probability of encountering asymmetry between sides in any given individual was 69%, while the probability that the left side would be more highly pneumatized than the right was 53%. The frequency of individual variations tracked in the APPS score are shown in Table 5.

**Table 4.** Paranasal Sinus Pneumatization According to Laterality

Side	APPS Score, mean ± SD	*P* Value
Left	4.95 ± 1.77	0.006
Right	4.74 ± 1.68	
More pneumatized side	5.35 ± 1.64	<0.001
Less pneumatized side	4.33 ±1.66	

APPS, Assessment of Pneumatization of the Paranasal Sinuses.

**Table 5.** Frequency of Individual Paranasal Sinus Anatomic Variations

Item/Anatomic Variant	Left Prevalence (n=323)	Right Prevalence (n=323)	Total Prevalence (n=646)	*P* Value, Right vs Left
1. Maxillary floor inferior to nasal floor	263 (81.4)	267 (82.7)	530 (82.0)	0.680
2. Supraorbital cell (air cell superior to anterior ethmoid artery)	80 (24.8)	76 (23.5)	156 (24.1)	0.777
3. Middle turbinate concha bullosa present	98 (30.3)	89 (27.6)	187 (28.9)	0.488
4. Frontal sinus present	310 (96.0)	307 (95.0)	617 (95.5)	0.572
5. Superior frontal sinus wall superior to supraorbital rim	282 (87.3)	274 (84.8)	556 (86.1)	0.362
6. Lateral frontal sinus wall lateral to medial edge of globe	259 (80.2)	254 (78.6)	513 (79.4)	0.624
7. Lateral frontal sinus wall lateral to midpupillary line	85 (26.3)	61 (18.9)	146 (22.6)	0.024
8. Lateral sphenoid sinus wall lateral to V_2_-VN line	172 (53.3)	145 (44.9)	317 (49.1)	0.034
9. Anterior clinoid process pneumatized	49 (15.2)	57 (17.6)	106 (16.4)	0.396

Note: Data are presented as n (%).

V_2_, maxillary nerve canal; VN, Vidian nerve canal.

## DISCUSSION

Wide variation in paranasal sinus pneumatization appears to exist, and the mechanisms for these differences are poorly understood. While certain sinonasal or respiratory pathologies are associated with measurable changes in sinus pneumatization, these variations are not restricted to a diseased state.^13-15^ More than 50 variations of the bony and air cell structure of the paranasal sinuses have been described, and up to 40% may not correspond with disease presentation.^15^ Furthermore, pervasive differences in paranasal sinus pneumatization, according to parameters other than clinical disease, continue to be an area of investigation among anatomists and clinicians.^22^ Understanding the degree of variation in nondiseased patients might better contextualize aberrations seen in the setting of clinical disease and may also help in the development of standardized sinus CT reports to aid clinician interpretation of the relevant anatomy.

Global paranasal sinus pneumatization seems to differ between males and females, with male subjects having increased pneumatization (Table 3). The APPS score, used to estimate total sinus volume in this study, also tracks individual anatomic variations and showed a decreased number of pneumatization variants in females. These findings are consistent with other reports of decreased volume of individual sinuses in females.^23-26^ Maxillary and frontal sinus volume on CT have even been reported to successfully differentiate between males and females in forensic analysis.^24-26^

In the current study, the male group was statistically younger than the female group, which may confound the finding of increased sinus pneumatization in males. We found no correlation, however, of age with the pneumatization of developed sinuses (Figure 2). As stated previously, all patients in this study were 13 years or older, at which point pneumatization was presumed to be complete.^20^ Reports of age-related pneumatization differences after complete sinus development conflict.^23,27^ Overall, our study suggests that among nondiseased patients, males have increased paranasal sinus pneumatization, and age-related differences are not present in completely developed sinuses.

Paranasal sinus morphology appears to remain stable across ethnic groups despite frequent variation among individuals.^28^ However, clinical sinus disease such as chronic rhinosinusitis remains an important health concern among different races and ethnicities.^29^ Investigations of anatomic differences according to ethnicity have been mixed. Some studies have demonstrated differences of particular variants and total sinus volume between specific ethnic groups,^28,30^ while others have found a similar degree of anatomic variation between ethnicities.^31^ Our study did not detect differences in overall paranasal sinus pneumatization between patients of Caucasian, African American, or Latinx/Hispanic ethnicity. Conclusions regarding ethnicity-related differences, however, are limited secondary to the small number of Latinx/Hispanic patients included in this study.

Comparisons of overall paranasal sinus pneumatization according to laterality have not been reported previously. In our study, increased pneumatization on the left side reached statistical significance (Table 4). Analysis of the individual variations tracked in the APPS score indicated that pneumatization lateral to a line drawn from the maxillary nerve canal to the Vidian nerve canal and frontal sinus pneumatization lateral to the midpupillary line were more frequent on the left (Table 5). Although statistically significant, the mean difference in APPS score between the right and left sides was small at 0.21 and may not have clinical significance. Alternatively, this difference could indicate selective pressures that influence laterality-based development in the head and neck. Embryologic mechanisms for laterality differences of anatomic structures in the head and neck, such as the course of recurrent laryngeal nerve and thoracic duct, are known. Similar mechanisms for sinonasal structures have been incompletely studied. Regardless of the specific side, the number of increased pneumatization variants tended to track together. The number of variants on the larger side was statistically greater than those on the smaller side, and the 2 sides were more frequently asymmetric. In 69% of cases, the paranasal sinus pneumatization would be expected to exhibit perceptible asymmetry. Moreover, in 53% of cases, the left side would be expected to be perceptibly more well pneumatized, while 47% would be either symmetric or more well pneumatized on the right.

This study has several limitations. First, numerous conceivable paranasal sinus variants were not included for analysis, which might influence the interpretation of the number of variants when comparing the different groups in this study. The International Frontal Sinus Anatomy Classification (IFAC) system was introduced shortly after the APPS score^32^ and was subsequently validated for rater reliability.^33^ Therefore, variants described in the IFAC system are not included in the present study. Nevertheless, the variants tracked in the APPS score can predict the total sinus volume^18^ and have also been validated for interrater and intrarater reliability.^17^ The features of the APPS instrument allow for the simultaneous tracking of individual anatomic variations in addition to a global assessment of paranasal sinus pneumatization. Second, the cross-sectional design of this study limits comment on changes in sinus anatomy over time. These changes may even occur in nondiseased patients, and if so, would influence interpretation of anatomic findings in the setting of sinus disease. As mentioned previously, the male group was statistically younger than the female group which may confound these results, and the small number of Latinx/Hispanic patients in this study may limit generalizable conclusions about ethnicity as a factor in sinus pneumatization.

## CONCLUSION

Patients without sinus disease have considerable variation in paranasal sinus anatomy. CT remains the standard for the radiographic evaluation of sinus disease. Our evaluation of sinus CTs using a validated metric revealed pervasive differences in overall sinus pneumatization between males and females and according to laterality, with the probability of encountering perceptible asymmetry approaching 70%. We did not detect differences across ethnic groups or according to age. Improved understanding of baseline anatomic paranasal sinus variation may facilitate standardized assessment of sinus CT and aid clinician anatomical interpretation.

## Notes

This article meets the Accreditation Council for Graduate Medical Education and the American Board of Medical Specialties Maintenance of Certification competencies for Patient Care and Medical Knowledge.
